# Antibacterial Ti-Mn-Cu alloys for biomedical applications

**DOI:** 10.1093/rb/rbaa050

**Published:** 2020-12-03

**Authors:** Mohammad Alqattan, Linda Peters, Yousef Alshammari, Fei Yang, Leandro Bolzoni

**Affiliations:** The University of Waikato, Hamilton, New Zealand

**Keywords:** titanium alloys, powder metallurgy, homogeneous microstructure, mechanical properties, antibacterial activity, *E. coli* (*Escherichia coli*)

## Abstract

Titanium alloys are common biomedical materials due to their biocompatibility and mechanical performance. However, titanium alloys are expensive and, unless surface treated, generally cannot prevent surgical infections related to bacteria which can damage the integrity of the implant. In this study, new titanium alloys were developed via powder metallurgy and the addition of manganese and copper, respectively, aiming to limit the manufacturing costs and induce new functionality on the materials including antibacterial response. The addition of manganese and copper to titanium significantly changes the behaviour of the Ti-Mn-Cu alloys leading to the successful stabilization of the beta titanium phase, great refinement of the typical lamellar structure, and achievement of materials with low level of porosity. Consequently, it is found that the mechanical performance and the antibacterial efficacy are enhanced by the addition of a higher amount of alloying elements. The manufactured Ti-Mn-Cu alloys fulfil the requirements for structural biomedical implants and have antibacterial response making them potential candidates for permanent medical implants.

## Introduction

Titanium and the Ti-6Al-4V alloy are common biomedical materials, respectively, used for dental applications and total joint replacement prosthesis [1, 2]. This is because, despite being bioinert, Ti alloys have good biocompatibility with the human body, environmental stability when in contact with human fluids due to the TiO_2_ passivation layer present in the surface of the material, and adequate level of mechanical strength combined with a relatively low Young modulus. Specifically, titanium alloys have a Young modulus more compatible with that of human bones with respect to other metallic materials used in biomedicine [1, 3]. However, there are different aspects that still limit the efficacy of titanium alloys for biomedical application. These are cytotoxicity of some of the alloying elements commonly used, stress shielding effect [4], high cost and inability to prevent antibacterial infection during implant surgery and during the early stages of recovery.

Cytotoxicity [5–8] and neurotoxic effects [6] of the alloying elements of the Ti-6Al-4V alloy are well documented in the literature and it is induced by the release of the alloying elements metallic ions. Therefore, aluminium and vanadium should, if possible, be avoided in the formulation of human body friendly materials for biomedical applications.

The poor transfer of the mechanical loads that the human body experiences while performing physical activities (e.g. walking) from the metallic prosthesis to the human bones (i.e. stress shielding) is due to the Young modulus mismatch. To limit this effect, beta titanium alloys [9] such as the Ti-13Zr-13Nb [10] and TNTZ (Ti-Nb-Ta-Zr) [11] alloys have been proposed. These alloys have however manufacturing limitations due to the high melting point of the refractory metals used as alloying elements and they are more expensive than common titanium alloys as their compositions entail large amount of costly and not widely available and sustainable elements.

Concerning the high cost of titanium, this derives from both extraction and manufacturing. A comprehensive strategy to make titanium more affordable will concurrently aim to limit the cost of the material using cheap alloying elements and reduce the manufacturing costs using more cost-effective techniques. Powder metallurgy is a sustainable green technology ideally suited to manufacture titanium alloys at reduced cost as products with the final shape can easily be obtained through few highly effective manufacturing steps [12]. In particular, the blended elemental approach where powders of the individual elements are mixed together gives the possibility to easily design and modify new chemical compositions. When combined with cold uniaxial press and sinter, the most basic of the powder metallurgy techniques, minimization of manufacturing cost is achieved [13].

Bacterial infection in biomaterials such as Ti-6Al-4V during surgical implantation and in the early hours of recovery is still a major limitation to be overcome [14]. This is one of the primary causes of biomedical implants failure [15] because infection associated with inflammatory reactions hinders the formation of new bone around the surgical implant [16]. To prevent bacterial infection, the addition of copper to either titanium or titanium alloys has been investigated. Specifically, Liu et al. [17] proved that cast Ti-Cu alloys for dental implants have superior capability to inhibit bone resorption derived by bacterial infection and inhibit biofilm formation in peri-implants without compromising the corrosion resistance with respect to titanium. The addition of copper to standard titanium compositions was studied considering the ball milled Ti-5Al-2.5Fe alloy where the addition of 1–5 wt.% of copper effectively prevented the spreading of *Escherichia coli* and *Staphylococcus aureus* bacterial colonies maintaining a cytotoxicity level within the standard range of values [18]. The addition of copper to the cast Ti-6Al-4V alloy has also been analysed and the mechanical behaviour quantified [19].

The current research addresses the development of Ti-Mn-Cu alloys produced via powder metallurgy (cost-effective manufacturing) for biomedical applications where the alloying elements (i.e. manganese and copper) where chosen because of their low cost, to limit the cost of the material, ability to improve the mechanical properties of titanium via the formation of two-phase microstructures, and interaction with the human body. Specifically, manganese promotes bone growth [20] (where powder metallurgy Ti-Mn alloys were previously studied [21]), copper stabilizes metabolic process [22] and provides antibacterial ability [17–19], and both of them induce bone induction and osteoblast reproduction [23]. The microstructure, mechanical properties and *in vitro* antibacterial efficacy of the Ti-Mn-Cu alloys were consequently studied.

## Experimental procedure

**Figure 1.**
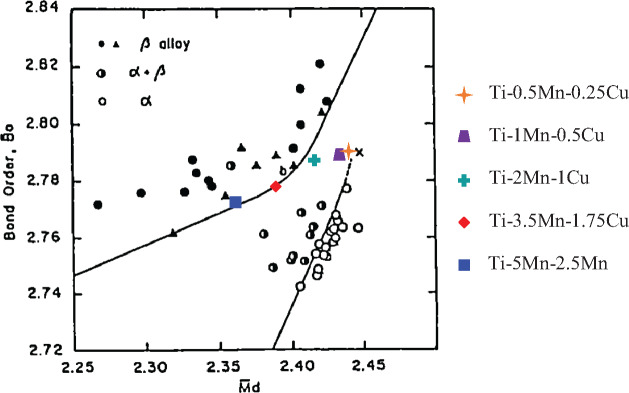
Phase diagram showing the expected type of Ti alloy, for the Ti-Mn-Cu alloys, as a function of the molecular orbital calculation of electronic structures (adapted from [25]).

Three elemental powders including an irregular Ti powder with particle size lower than 75 µm (99.4% purity), a dendritic Cu powder with particle size lower than 63 µm (99.7% purity), and an angular Mn powder with particle size lower than 45 µm (99.0% purity) were used for formulate the Ti-Mn-Cu alloys. The actual compositions, along with their density (which increases with the progressive addition of more alloying elements), are reported in Table 1 where it can be seen that the maximum amount of Mn and Cu is 5 wt.% and 2.5 wt.%, respectively. The amount of each alloying element was chosen using the respective phase diagrams [24] to maximize the reduction of the cost but aiming to prevent the formation of brittle phases such the athermal omega phase typical of binary Ti-Mn alloys [21] and other intermetallic phases that would compromise the ductility of the alloy. The chemistry of the alloys was designed as per the theoretical framework proposed by Morinaga et al. [25] on the basis of the molecular orbital calculation of electronic structures. From Figure 1, which shows a phase diagram indicating the expected type of alloy, it can be seen that the Ti-Mn-Cu alloys are designed to be casi-alpha and alpha±beta Ti alloys (Table 1).

**Table 1.** Chemical composition, density and alloy type of the Ti-Mn-Cu alloys

Ti-Mn-Cu alloys	Density [g/cm^3^]	Alloy type
Ti-0.5Mn-0.25Cu	4.54	casi-α
Ti-1Mn-0.5Cu	4.56	α + β
Ti-2Mn-1Cu	4.61	α + β
Ti-3.5Mn-1.75Cu	4.69	α + β
Ti-5Mn-2.5Cu	4.77	α + β

A V-shape blender run at 45 Hz for 30 minutes was used to produce homogeneous powder blends for their pressing into cylindrical samples applying 600 MPa of uniaxial pressure at room temperature. Samples were subsequently heated at 10°C per minute to perform isothermal sintering at 1300°C for 2 hours under vacuum (10^−5^ mbar).

Analysis of the phases and of the microstructure was, respectively, done by XRD using a Panalytical X’pert equipment (30°–70° range using an increment of 0.013°) and microscopy for which the samples were previously prepared via the classical metallographic route. A 4 vol.% HF and 5 vol.% HNO_3_ aqueous solution was used for revealing the phases. Mass-to-volume ratios and Archimedes measurements were, respectively, used to quantify the density of the green and sintered samples. Dividing the obtained values by the respective density of the alloys reported in Table 1 allowed calculating the amount of residual porosity present in the specimens.

The hardness of the Ti-Mn-Cu alloys was obtained via HRA measurements. Tensile test pieces were cut from the original cylindrical samples to quantify the tensile behaviour. The test pieces (2 × 2 × 20 mm^3^) were pulled at 5 × 10^−3^ s^− 1^ and a mechanical extensometer was used to measure the deformation. For the mechanical characterization, at least three specimens were tested.

Culture tubes with *E. coli* culture streaked on aseptic agar plates with Luria Broth (Life Technologies Ltd.) were prepared for the *in vitro* antibacterial tests. Triplicate 5-μL serial dilution spotting method [10] for bacteria medium and serial dilutions (10^−2^ to 10^−8^) of overnight bacterial culture (OD_600_ = 0.8) were used to quantify the colony-forming units per millilitre (CFU). The viable number of bacteria colonies was in the 1–30 colonies range. *E. coli in vitro* antibacterial ability and efficacy was quantified using the plate count method as per [26] using 30 μL of inoculum. The CFU of the extracted bacteria was quantified considering as 30–300 per common 9-cm diameter agar dish [26, 27] after serial dilution to 10^−6^ by means of a standard PBS solution. The antibacterial reduction efficacy was quantified by means of the CFU values of the samples and of the reference (100×(1 − CFU_samples_/CFU_reference_)).

## Results and discussion

**Figure 2.**
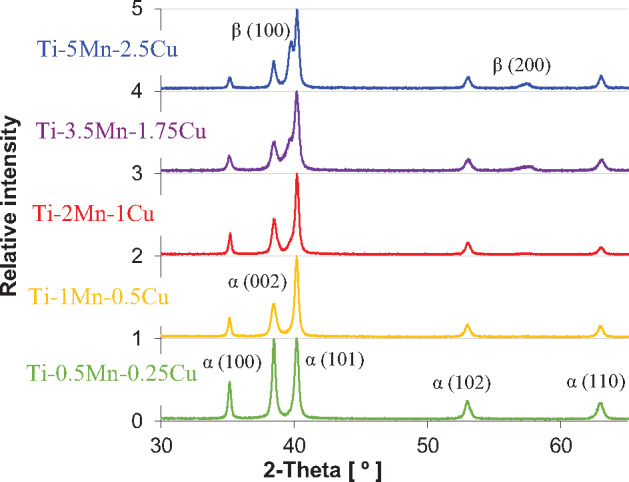
XRD diffractograms of the sintered Ti-Mn-Cu alloys.

Figure 2 shows the XRD diffractograms of the Ti-Mn-Cu alloys where it can be seen that the alpha titanium phase is the primary phase constituting the alloys. The relative intensity of the beta titanium phase increases with the addition of a higher amount of alloying elements as both manganese and copper are elements that stabilize the beta titanium phase. Consequently, the relative amount of beta titanium phase present in the microstructure of the Ti-Mn-Cu alloys is expected to be greater for more heavily alloyed compositions. However, the main peak corresponding to the beta titanium phase is not detected in lean alloyed compositions as the amount of stabilise beta titanium phase is below the detection limit of the equipment. Conversely, the (200) peak of the beta titanium phase is also detected for more heavily alloyed compositions. It is worth noticing that, generally, no secondary phases such as the athermal omega titanium phase, which was reported for Mn addition greater than 8–9 wt.% [21], manganese-based intermetallic compounds, or the copper-based Ti_2_Cu intermetallic compound are detected independently of the alloy composition, as they could be expected on the basis of the phase diagrams [24].

**Figure 3.**
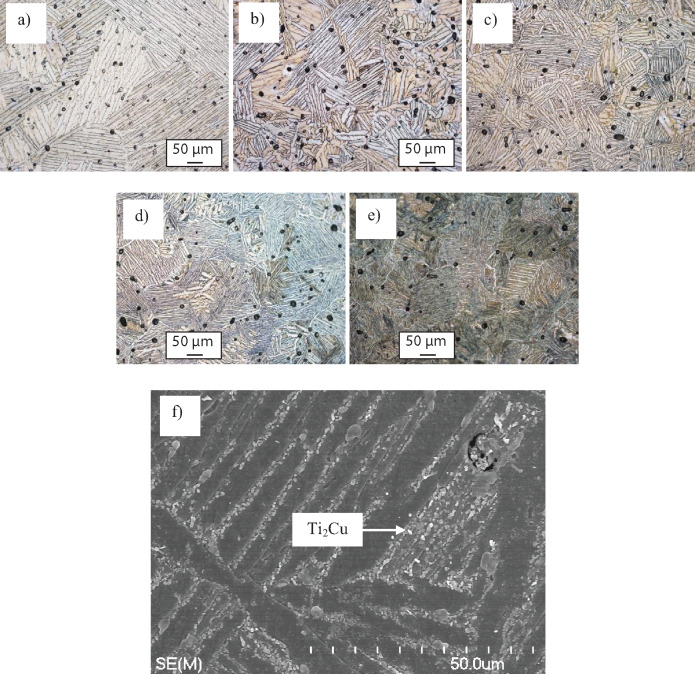
Optical micrographs of the sintered Ti-Mn-Cu alloys showing the microstructural features present (phases and porosity). (a) Ti-0.5Mn-0.25Cu, (b) Ti-1Mn-0.5Cu, (c) Ti-2Mn-1Cu, (d) Ti-3.5Mn-1.75Cu and (e) Ti-5Mn-2.5Cu; and f): SE-SEM micrograph of the Ti_2_Cu intermetallic compound precipitated at the at the lamellar boundaries in the Ti-5Mn-2.5Cu alloy.

The results of the microstructural analysis are shown in Figure 3 where it can be seen that, irrespective of the chemistry of the alloy, the sintered Ti-Mn-Cu alloys have a fully lamellar microstructure composed of nearly equiaxed alpha titanium grains and parallel alpha titanium and beta titanium lamellae. As the content of manganese and copper increases, the relative amount of beta titanium phase present in the microstructure increases linearly. It is worth noticing that the fineness of the lamellae increases, and therefore the distance between them is reduced, for higher contents of manganese and copper (Figure 3a–e). As these microstructural features control the mechanical behaviour of the alloy, it is expected that the stabilization of a greater amount of beta titanium phase and the achievement of finer lamellae will lead to the increase of the strength and the reduction of the ductility as the movement of the dislocations will be progressively more significantly hindered.

**Figure 4.**
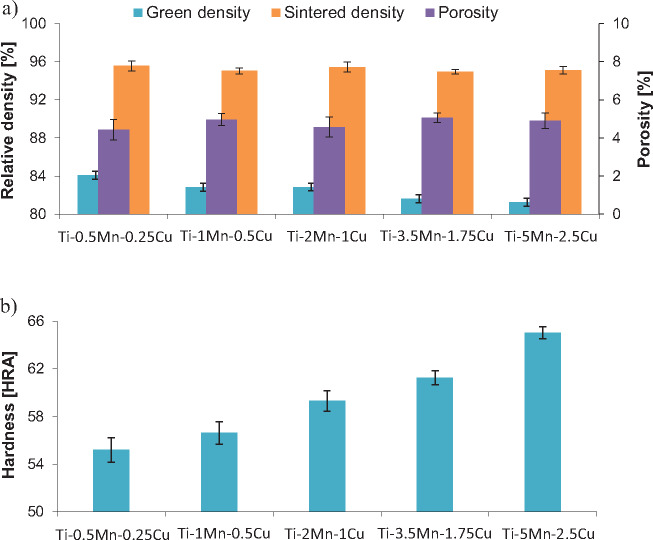
Values of the density/porosity (a) and of the hardness (b) of the sintered Ti-Mn-Cu alloys.

Selection of the sintering temperature to achieve Ti-Mn-Cu alloys with homogenous chemistry was done on the basis of the literature about blended elemental Ti alloys at 1300°C for 2 hours [28, 29], which was confirmed via EDS analysis. For the Ti-5Mn-2.5Cu alloys, which has the highest amount of alloying elements that need to diffuse to obtain a homogeneous composition, through EDS it was found an average chemical composition of 92.31Ti-4.59Mn-3.10Cu where the detected contents of manganese and copper are, respectively, slightly lower and higher than the theoretical values due to their atomic number. As both manganese and copper are beta stabilizing elements with fairly limited solubility in the alpha titanium phase, especially Mn [24], the alloying elements are primarily distributed and found in the beta titanium lamellar phase. Moreover, for the Ti-5Mn-2.5Cu alloy, precipitation of the Ti_2_Cu intermetallic compound at the lamellar boundaries between the alpha titanium and beta titanium phases occurred (Figure 3f). Microstructural analysis also shows that some residual porosity is present within the microstructure of the sintered Ti-Mn-Cu alloys where the total amount of porosity is almost constant with the content of manganese and copper added to Ti (see Figure 4). However, the majority of the pores are spherical in shape and are uniformly distributed within the microstructure. The presence of these pores has an impact on the mechanical behaviour of the alloys as pores are weak points of the structure and most likely the *loci* where failure of the material would preferentially initiate.

The values of the density of the sintered Ti-Mn-Cu alloys, including values of the density of the green and sintered specimens as well as the porosity, are represented in Figure 4. The green density slightly decreases (84%–81%) with the progressive addition of more alloying elements due to the mismatch between the morphology of the starting powders as well as their hardness and deformability. In terms of sintered density, and in turn porosity, the alloying elements content has minor influence on the final value which is almost constant at 95 ± 0.5%. This means that the slight difference in compressibility of the powder mixtures is levelled out by the effect of the alloying elements during the densification of the alloy, where Mn promotes densification as it has been found in binary Ti-Mn alloys produced by powder metallurgy [21]. Accordingly, the level of residual porosity obtained via water displacement measurements is nearly constant 5%, value which is consistent with the microstructural characterization presented in Figure 3. It is worth mentioning that the chemistry of the alloy does not have a statistically relevant influence on the oxygen content of the sintered Ti-Mn-Cu alloys, whose average value is 0.27 ± 0.04 wt.%.

The variation of the HRA hardness of the sintered Ti-Mn-Cu alloys is shown in Figure 4b where it can be seen that the hardness increases from 55 HRA to 65 HRA as the content of manganese and copper increases. As the sintered Ti-Mn-Cu alloys have similar amount of residual porosity, the increase of the hardness is related to the greater amount of stabilised beta titanium phase, the formation of the lamellar structure (Figure 3), and the solid solution strengthening induced by the dissolution of manganese and copper into the titanium lattice. Although the Ti_2_Cu intermetallic compound was detected in the Ti-5Mn-2.5Cu alloy, this phase does not significantly increase the hardness with respect to the Ti-3.5Mn-1.75Cu alloy. This means that the amount of Ti_2_Cu intermetallics is low (i.e. not detected via XRD analysis, Figure 2) and their size is very small (Figure 3f).

**Figure 5.**
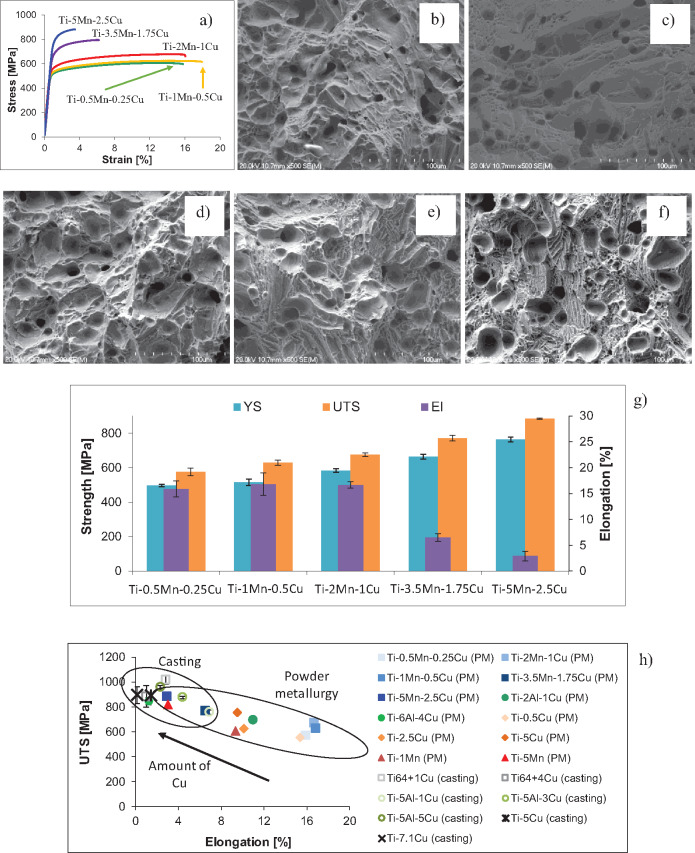
Representative tensile behaviour of the sintered Ti-Mn-Cu alloys: (a) stress-strain curves, fracture surface of the (b) Ti-0.5Mn-0.25Cu, (c) Ti-1Mn-0.5Cu, (d) Ti-2Mn-1Cu, (e) Ti-3.5Mn-1.75Cu and (f) Ti-5Mn-2.5Cu alloys, (g) average tensile properties and (h) comparison with other antibacterial Ti-based alloys [19, 30–34].

The results of the characterization of the tensile behaviour of the sintered Ti-Mn-Cu alloys are reported in Figure 5. The stress–strain curves show that the sintered Ti-Mn-Cu alloys have both elastic and plastic behaviour and the transition point between the elastic and plastic region is gradually shifted to higher applied loads as more manganese and copper are added to titanium. The Ti-0.5Mn-0.25Cu and Ti-1Mn-0.5Cu alloys have comparable UTS but the Ti-1Mn-0.5Cu alloy has slightly higher elongation at fracture despite the greater amount of alloying elements because of its better balance between porosity and characteristics of the phases present in the microstructure.

In agreement with the tensile curves, the fracture mode of the sintered Ti-Mn-Cu alloys changes from purely ductile, with a fracture surface composed of large dimples of roughly the size of the alpha titanium grains, to a combined ductile/brittle fracture mode (Figure 5). Precisely, the refinement of the lamellae induced by the incremental addition of more manganese and copper, leads to some transgranular fracture within the lamellar structure, generation of river-like pattern within the alpha titanium grains as well as some tear-ridges between these alpha titanium grains for the Ti-5Mn-2.5Cu alloy (Figure 5f).

The values of the average tensile properties reinforce the findings from the stress–strain curves as both yield strength and ultimate tensile strength linearly increase with the addition of more manganese and copper, while the average elongation remains constant around 16% up to the Ti-2Mn-1Cu and then decreases as more alloying elements are added as the alloys have comparable level of residual porosity and oxygen content. From Figure 5h, for similar amount of copper or manganese, the sintered Ti-Mn-Cu alloys have generally better UTS vs. elongation than the respective binary Ti-Mn and Ti-Cu powder metallurgy alloys. When the amount of alloying elements is relatively low, the sintered Ti-Mn-Cu alloys have lower strength but much higher ductility with respect to other antibacterial Ti-based alloys, regardless of the manufacturing route. The Ti-3.5Mn-1.75Cu alloy has very similar properties to the cast Ti-5Al-1Cu alloy [34], whereas the Ti-5Mn-2.5Cu alloys has similar strength but better elongation than cast binary Ti-Cu alloys [19, 33]. Generally, the alloys obtained via powder metallurgy have slightly lower strength but much better ductility than the cast alloys. Finally, the higher the amount of copper (or alloying elements in general) the harder and the more brittle the material.

**Figure 6.**
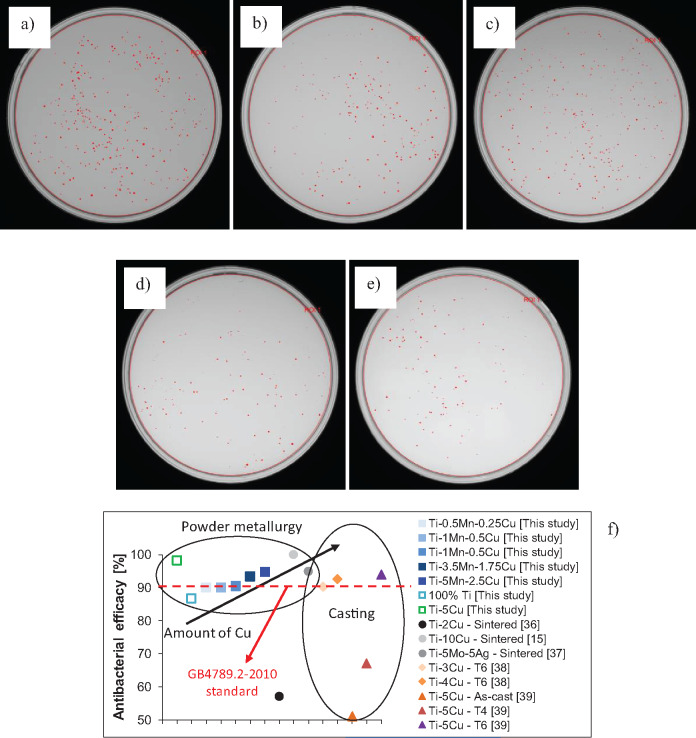
Digital images of the *E. coli* colonies found on the surface of the sintered Ti-Mn-Cu alloys: (a) Ti-0.5Mn-0.25Cu, (b) Ti-1Mn-0.5Cu, (c) Ti-2Mn-1Cu, (d) Ti-3.5Mn-1.75Cu, (e) Ti-5Mn-2.5Cu and (f) comparison of the antibacterial efficacy with literature [36–39].

The results of the *in vitro* antibacterial efficacy of the sintered Ti-Mn-Cu alloys are presented in Figure 6. As the amount of manganese and copper added to titanium increases, the quantity of *E. coli* colonies present once elapsed the 24-h inoculation period decreases as visible from the digital images. Specifically, it can be seen that due to the low amount of alloying elements, the antibacterial efficacy of the Ti-0.5Mn-0.25Cu, Ti-1Mn-0.5Cu and Ti-2Mn-1Cu alloys is very similar. For these three materials, the antibacterial efficacy is just above 90%, and therefore they can be considered materials with antibacterial ability as per the GB4789.2-2010 Food Safety Standard [35]. Although their antibacterial efficacy is not as high as that of the sintered Ti-5Cu alloy, the antibacterial efficacy of the Ti-3.5Mn-1.75Cu and Ti-5Mn-2.5Cu alloys is superior to that of the other sintered Ti-Mn-Cu alloys.

From the comparison of the antibacterial efficacy results of the sintered Ti-Mn-Cu alloys with respect to available literature [36–39], it is found that the sintered Ti-Mn-Cu alloys have mainly comparable antibacterial efficacy to that of other copper-bearing titanium alloys (Figure 6). More in detail, regardless of the manufacturing process, the antibacterial efficacy increases with the amount of copper where the highest values are obtained for the Ti-5Cu and Ti-10Cu alloys. Most of the copper-bearing alloys have better antibacterial efficacy than pure titanium with the exception of the sintered Ti-2Cu alloy, as-cast Ti-5Cu alloy, and heat treated (T4) Ti-5Cu alloy, which have antibacterial efficacy lower than 70% and, thus, cannot be considered as antibacterial materials on the basis of the GB4789.2-2010 Food Safety Standard [35]. The performance of the copper-bearing alloys is similar to that of the Ti-5Mo-5Ag alloys, which is much more expensive due to the presence of costly alloying elements. Considering the manufacturing route, the sintered Ti-Mn-Cu alloys have better antibacterial efficacy right after their main manufacturing process (i.e. sintering), whereas the cast copper-bearing alloys need to be heat treated to be able to reach antibacterial efficacy values above 90%.

## Conclusions

This study demonstrates that Ti-Mn-Cu alloys can successfully be synthesized using the blended elemental powder metallurgy approach and they can easily be manufactured via the cold press and sinter route. From the characterization of the behaviour of these novel alloys, the addition of manganese and copper to titanium leads to the stabilization of the beta titanium phase, the formation of a progressively refined lamellar structure with finer lamellae and interlamellar spacing, fairly constant level of residual porosity, and successful prevention of the formation and precipitation of secondary phases which will embrittle the material. Accordingly, the addition of a higher amount of manganese and copper monotonically increases the resistance of the alloys against quasi-static loadings, progressively increases the antibacterial efficacy, but eventually leads to the reduction of the ductility and, thus, the ability of the alloy to withstand permanent deformation. Based on the assessment of the mechanical performance and antibacterial response, the Ti-Mn-Cu alloys are potential candidates for permanent medical implants. 
